# Development of Chinese chestnut whiskey: yeast strains isolation, fermentation system optimization, and scale-up fermentation

**DOI:** 10.1186/s13568-020-01175-4

**Published:** 2021-01-11

**Authors:** Wanzhen Li, Cuie Shi, Jiaquan Guang, Fei Ge, Shoubao Yan

**Affiliations:** 1 College of Biological and Food Engineering, Anhui Polytechnic University, Anhui, 241000 People’s Republic of China; 2 School of Life Science, Huainan Normal University, Huainan, Anhui 232001 People’s Republic of China; 3 Anhui Yingjia Group Co., Ltd, Luan, Anhui Province 237271 People’s Republic of China

**Keywords:** Chinese chestnut, Whisky, Volatile flavor compounds, Fermentation

## Abstract

In this study, we used Chinese chestnut as the main raw material to develop a novel type of whiskey. First, 16 yeasts were isolated and identified for producing aroma using olfactory plate assay. Of these, we screened nine yeast strains based on their fermentation capacity, aroma profile, and sensory evaluation. The results demonstrated the combination of strains HN006 (*Saccharomyces cerevisiae*) and HN010 (*Wickerhamomyces anomalus*) provided satisfactory wine fermentation with an interesting flavor profile, as strain HN010 was highly aromatic and had elevated sensory scores with comparatively low ethanol yield, while strain HN006 had a poor flavor profile but produced the largest amount of ethanol. Subsequently, we co-cultured strains HN006 and HN010 to optimize the fermentation system. The results revealed the following optimum parameters: a mixed inoculum of 6% (v/v) at an HN006/HN010 ratio of 1:2 (v/v), a raw material ratio of 5:3:2 (chestnut: malt: glutinous rice), and yeast extract concentration of 6 g/L. Additionally, this fermentation system was successfully scaled-up to a 1000 L pilot-scale system. The results of this study showed that strains HN006 and HN010 could be used as alternatives for whiskey fermentation, as well as provided a generalized experimental scheme to assess other microorganisms.

## Introduction

Chinese chestnut, one of the major nuts in China, belongs to the genus *Castanea* *Miller* and the family *Fagaceae* and is also known as “the king of dry fruit” (Zhang et al. 2018a, b). Its edible kernels are popular in China and are rich in both macronutrients (starch, protein, and fat) and micronutrients (minerals, such as iron, calcium, phosphorus, and several types of vitamins, as well as trace minerals). Modern pharmacological studies have shown that the Chinese chestnut nourishes the stomach and the spleen, improves the functioning of kidneys, strengthens the tendons, activates blood circulation, and is used for the prevention and treatment of hypertension and coronary heart disease (Zhang et al. 2014).

Chinese chestnut is a cultivated plant with a history dating back approximately 3000 years in China, and ever since, there has been an annual increase in the production of chestnuts (Zhang et al. 2018a, b). However, the phenomenon of "difficult to sell chestnut" still exists in many planting areas in China, which has reduced the motivation of the chestnut farmers. Additionally, the seasonal availability of Chinese chestnut fruits along with their high water content (approximately 50% on a wet basis) limits their storage due to germination, mildew, and insect infestation (Sheng and Yu 2015). Currently, the main processing methods of Chinese chestnuts include baking, cooking, or frying with sugar (tang chao li zi), which is not beneficial economically. Thus, there is an urgent need to identify and develop novel processing methods for using Chinese chestnuts to produce high-value products, such as Chinese chestnut wine.

Whiskey is one of the four famous distilled wines in the world and is popular internationally. The most famous whiskey producing countries are Scotland, Ireland, the USA, Japan, and Canada. However, there are few reports on whiskey production in China. The high starch content of the Chinese chestnut makes it suitable for whiskey brewing. However, Chinese chestnut whiskey (wine) is still scarce in the domestic and foreign markets.

The sensory attributes of whiskey result from compounds that originate from the raw materials, the metabolites of yeast, and maturation process. Among them, the yeast make a special contribution to the flavour quality of whiskey, and the selection of microorganisms is critical for the formation of aroma, which is one of the key attributes affecting consumer acceptance (Du et al. 2019).

Therefore, this study focused on developing a novel whiskey using the Chinese chestnut as the main raw material. We isolated and investigated the potential of specific yeast strains for effective Chinese chestnut wine fermentation. Additionally, we used the screened strains to optimize the fermentation process based on high ethanol yield and sensory scores. Furthermore, we used a 1000 L pilot-scale system to scale-up the fermentation to industrialize the process of Chinese chestnut wine production. The results of this study would provide supportive evidence to develop a Chinese chestnut-based novel whiskey.

## Materials and methods

### Materials

Chinese chestnuts and glutinous rice were purchased from a retail market in the Huoshan county, Anhui province of China, in October 2019. The malt was obtained from Zhengzhou Longhai beer Materials Co., Ltd (Zhengzhou, Henan province, China). The thermostable α-amylase and glucoamylase for saccharification were acquired from the Sunson Industry Group Co., Ltd. (China).

### Isolation of superior yeast strain

#### Initial screening for yeast strains

In a 250 mL flask, we mixed 5 g of Chinese strong flavour *Daqu,* which is a saccharifying and fermenting agent utilized in the brewing of Chinese strong flavour liquor and contains a variety of yeast species, into 100 mL of yeast extract peptone dextrose medium (YEPD; 5 g/L yeast extract + 50 g/L glucose + 10 g/L peptone). The mixture was stirred at 200 rpm at 30 °C for 24 h. The culture was serially diluted with sterile water and coated on the YEPD plates supplemented with 0.01% chloromycetin in duplicates. Next, 48–72 h later, we isolated colonies with varying morphologies and purified them, followed by inoculation on a YEPD agar plate to conduct a smelling or “sniffing” test for initial aroma screening following the method of Alicia et al. (2018).

### Repetitive element sequence-based PCR (Rep-PCR) analysis and yeast identification

A DNA extraction kit was used to extract the genomic DNA of the isolated yeast strains (Sangon Biotech, Shanghai, China). The rep-PCR analysis was done using the primer (GTG)_5_ (5′-GTGGTGGTGGTGGTG-3′) using a previously described method (Simonetta et al. 2016). A 1.2% agarose gel electrophoresis (AGE) was used to separate the amplicons in a 1 × TBE buffer (150 min, 120 V). The clusters were identified by analyzing the resultant dendrograms.

**Fig. 1:**
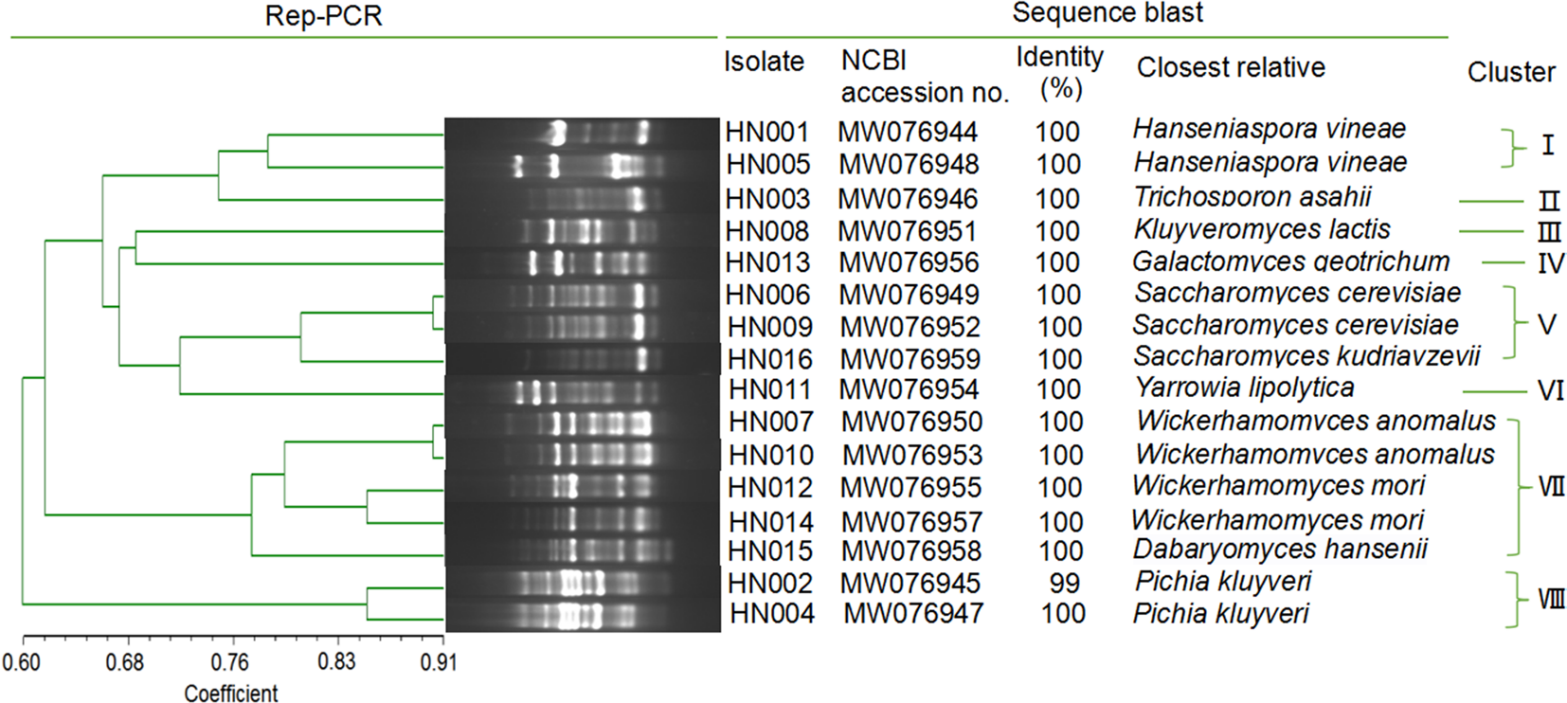
Dendrogram obtained by cluster analysis of (GTG)_5_-based rep-PCR fingerprints of the isolated yeasts, based on Dice’s coefficient of similarity with the unweighted pair group method with arithmetic average clustering algorithm (UPGMA). Yeast species were identified by sequencing of 26S rRNA gene and GenBank searches

For identification of the yeast, the universal primer pairs NL1 and NL4 were used to sequence the strain of each rep-PCR fingerprint group (Yan et al. 2019a, b), followed by alignment to the 26S rRNA gene sequences in the GenBank database using the BLAST algorithm tool. The nucleotide sequences obtained in this study have been assigned GenBank Accession Nos. MW076944-MW076959, as indicated in Fig. 1.

### Testing for the formation of volatile flavor compounds

Chinese chestnuts were peeled, cleaned, and steam-cooked for 30 min. After naturally cooling to room temperature, the cooked chestnut kernels were mixed with water (1: 3 (m/v)) and pulped using a fruit mixer. Next, the mixtures were transferred into a bioreactor and saccharified using α-amylase (10 U/g chestnut kernel; pH 5.5, 90 °C, 30 min) and subsequently, glucoamylase (200 U/g chestnut kernel; pH 4.5, 60 °C, 6 h). Then, a double gauze was used to separate the liquid phase of the saccharified mixture and placed into a 2.5 L glass bottle, each with 2 L volume. The liquid phase of the saccharified mixture was sterilized at 113 °C for 10 min and subjected to ethanol fermentation. The conditions of the fermentation and distillation of Chinese chestnut wine have been listed in Sect. 2.3. Finally, the volatile compounds in each sample of the obtained raw wine were detected and quantified by solid-phase microextraction-gas chromatography/mass spectrometry (SPME–GC–MS).

### Testing for fermentation ability of isolated strains

The seed colonies of each isolated yeast strain were cultured aerobically in liquid YEPD medium for 24 h at 30 °C with constant stirring at 200 rpm. Next, 5% (v/v) of these suspension cultures (approximately 1 × 10^7^ CFU/mL) were added to 250 mL Erlenmeyer flasks containing YEPD medium (200 mL) with 190 g/L glucose. The flasks had perforated silicon stoppers equipped with 0.45 mm filters (Merck Millipore, Italy), to release carbon dioxide and to prevent contamination. All the tests were carried out in triplicates and performed under static conditions at 30 °C. Post-fermentation, each fermentation broth was sampled to detect the remaining reducing sugar content, as well as the ethanol content.

### Sensory evaluation of the flavor of yeast fermentation products

Section 2.6 describes the methods used for the evaluation of the sensory features, including smell, taste, flavor, acidity, and overall acceptability of each obtained distillate. Further testing was done using yeast strains with high total scores.

### Laboratory fermentation of Chinese chestnut wine

Additional file 1: Figure S1 shows the manufacturing process of Chinese chestnut whiskey raw wine. The Chinese chestnut kernel, malt, and glutinous rice were cleaned and soaked (glutinous rice was soaked for 2 h in 1.5 times water). Post-soaking, glutinous rice and Chinese chestnut kernels were steam-cooked for 30 min and cooled to room temperature. Then, water was added at a ratio of 1: 3 (m/v) and the mixture was saccharified by α-amylase (10 U/g raw material) at pH 5.5, 90 °C for 30 min; followed by saccharification with glucoamylase at pH 4.5, 60 °C for 6 h with the addition of the pre-crushed malt. Subsequently, a double gauze was used to separate the liquid phase of the saccharified mixture, followed by adjusting the pH to 5.0 and transferring the contents into a 5 L fermenter with 4 L of working volume. After sterilization at 113 °C for 10 min, the prepared sugar solution was subjected to wine fermentation by inoculation with pre-cultured yeast seeds. The temperature of the fermentation system was maintained at 30 °C.

Post-alcoholic fermentation, we immediately double-distilled the fermented liquids in a 5 L Charentais potstill. When the alcohol concentration was less than 0.5% (w/v), we stopped the first distillation, resulting in an approximately 20% (v/v) distillate. During the second distillation, approximately one-third of the distillate was collected, resulting in a final alcohol concentration of 60% (v/v) in the final raw Chinese chestnut wine.

### Optimization of Chinese chestnuts wine fermentation

We performed anaerobic Chinese chestnut wine fermentation as described previously for 6 days to optimize the fermentation process. At the end of the process, we collected samples of fermentation broth to determine the ethanol and residual sugar content, as well as to evaluate the overall sensory features of the obtained wine. We also evaluated the impact of different inoculum sizes on the production of Chinese chestnuts wine. Table 2 shows the settings used for this analysis. Additionally, we examined the impact of different raw materials on the brewing of chestnut whiskey. The Chinese chestnut kernels, malt, and glutinous rice were prepared in various ratios (Table 3) and were subjected to the wine fermentation process following the previously described procedure. Furthermore, we also tested different supplementation amounts of nitrogen nutrient (yeast extract). All experiments were conducted in triplicates, and the results were expressed as the average of the three repetitions.

### The inoculum preparation for 1000 L pilot-scale fermentation

For inoculum preparation, the suspended cells of the obtained yeast from single colonies on YEPD agar plates were pre-cultured in 250 mL flasks containing 100 mL YEPD medium at 30 °C for 24 h. Then each of 20 mL cultures were transferred to five 500 mL flasks each containing 180 mL YEPD medium, and cultivated under the same conditions mentioned above, after which the entire volume of the five 500 mL-flasks was used to inoculate a 10 L fermentor (v/v, 10%). The culture broth from the 10 L fermentor was transferred to a 150L fermentor containing 100L of growth medium. Both the fermentation scales of 10 and 150 L were conducted at 30 °C, 300 rpm, and VVM of 4.0 for 24 h. Finally the culture broth from 150 L fermentor was used for the pilot-scale fermentation. The volumes to be inoculated were determined on the basis of cell counting under a light microscope with a Thoma Zeiss chamber.

### 1000 L pilot-scale fermentation

We performed the pilot scaling-up experiments based on the optimization conditions derived from laboratory experiments in a pilot-scale plant built at Anhui Yingjia Distillery Co., Ltd., located in Huoshan county, Luan City, China. The scale-up fermentation was performed in a 1000 L fermenter with a liquid loading capacity of 900 L. Additional file 1: Figure S2 shows a schematic diagram of the experimental apparatus used in this study.

### Analytical methods

During the fermentation process, we collected broth samples at specific time intervals, followed by 20-min centrifugation at 5000 *g* to remove the cellular debris. The 3, 5-dinitro-salicylic acid colorimetric method was used to determine the reducing sugar content (Miller 1959). The measurement of yeast cells and the ethanol content was performed following the method of Yan et al. (2013).

### Analyses of the volatile compounds

The HS–SPME–GC–MS was done to analyze the aroma profiles of the obtained wine samples, following a previously described method (Yan et al. 2019a, b). The volatile flavor compounds in the samples were identified by comparing the mass spectrum data with the NIST 05a MS database (Agilent Technologies, Inc., USA), and quantification was done based on the comparison of their peak intensity to that of the 2-octanol (internal reference).

Additionally, heatmap clustering was used to analyze the HS–SPME–GC–MS dataset to identify the differences in the fermentation profiles of different yeast strains. The analysis was done using R (programming language) Software (Annalisa et al. 2018).

### Sensory evaluation

We evaluated the sensory characteristics of smell, taste, acidity, after-taste, and overall acceptability of the final Chinese chestnuts wine following a previously reported method (Yan et al. 2019a, b). Wine samples (20 mL) were labeled with a randomly assigned 3-digit code and simultaneously served in ISO tasting glasses covered with plastic Petri dishes at room temperature.

## Results

### Preliminary screening of yeast strains

Post-morphological examination (both micro and macro), we identified 16 isolates that were growing on YEPD agar as yeast strains. The cluster analysis of the rep-PCR of these 16, based on a coefficient of similarity of 76%, resulted in eight clusters (Fig. 1). We identified the isolates in each cluster by sequencing the D1/D2 region of the 26S rRNA gene, followed by a BLAST search at GenBank (Fig. 1). Approximately 31.25% of the yeast isolates were found in cluster VII, which were identified as *Wickerhamomyces anomalus* (HN007, HN010)*, Wickerhamomyces mori* (HN012, HN015), and *Dabaryomyces hansenii* (HN014). Approximately 18.75% of the yeast isolates were found in cluster V and shared 100% homology with GenBank sequences of *Saccharomyces cerevisiae* (HN006, HN009) and *Saccharomyces kudriavzevii* (HN016). The cluster I and VIII isolates were unambiguously identified as *Hanseniaspora vineae* (HN001, HN005) and *Pichia kluyveri* (HN002, HN004), respectively. The remaining four clusters (II, III, IV, and VI) were identified as *Trichosporon asahii* (HN003), *Kluyveromyces lactis* (HN008), *Galactomyces geotrichum* (HN013), and *Yarrowia lipolytica* (HN011), respectively, each showing 100% homology to GenBank sequences.

We performed the olfactory “sniff” test on the YEPD medium plates to preliminarily determine the aroma producing ability of the obtained 16 yeast strains. Table 1 presents the results of this analysis. We found that different genera of the isolates presented various aromas, differentiated by different intensities of sourness, fruity, floral, cheese-like, and cream-like smells. *Saccharomyces cerevisiae* (HN006, HN009) and *Saccharomyces kudriavzevii* (HN016) produced different intensities of alcoholic aroma in the YEPD agar plates. Both *Dabaryomyces hansenii* (HN014), and *Yarrowia lipolytica* (HN011) produced characteristic cheese-like smells with intermediate strength. Each of the following strains: *Trichosporon asahii* (HN003), *Wickerhamomyces anomalus* (HN007, HN010), *Wickerhamomyces mori* (HN012, HN015), *Pichia kluyveri* (HN002, HN004)*,* and *Kluyveromyces lactis* (HN008) produced pleasant fragrance of pineapple fruit, apple and rose fruit, apple fruit, banana fruit, and strawberry fruit, respectively, and among which strain *Wickerhamomyces anomalus* (HN010) possessed the highest intensity. While the strain *Hanseniaspora vineae* (HN001, HN005) generated an unpleasant sour flavor, the strain *Galactomyces geotrichum* (HN013) produced a cream-like aroma, which was reported to have wide application in the fermentation of dairy products (Jacques et al. 2017).

**Table 1:** The pleasant aroma characteristics of the obtained strains by agar plate “sniff” assay method

Strain code	Closest relative^1^	Aroma	Aroma intensity^2^
HN001	*Hanseniaspora vineae*	Sour aroma	+
HN002	*Pichia kluyveri*	Banana fruity aroma	+ +
HN003	*Trichosporon asahii*	Pineapple fruity aroma	+ +
HN004	*Pichia kluyveri*	Banana fruity aroma	+
HN005	*Hanseniaspora vineae*	Sour aroma	+
HN006	*Saccharomyces cerevisiae*	Alcohol aroma	+ +
HN007	*Wickerhamomyces anomalus*	Apple fruity aroma	+ +
HN008	*Kluyveromyces lactis*	Strawberry fruity aroma	+ +
HN009	*Saccharomyces cerevisiae*	Alcohol aroma	+
HN010	*Wickerhamomyces anomalus*	Apple fruity and rose aroma	+ + +
HN011	*Yarrowia lipolytica*	Cheese and sweet aroma	+ +
HN012	*Wickerhamomyces mori*	Apple fruity aroma	+
HN013	*Galactomyces geotrichum*	Cream aroma	+ +
HN014	*Dabaryomyces hansenii*	Cheese aroma	+ +
HN015	*Wickerhamomyces mori*	Apple fruity aroma	+
HN016	*Saccharomyces kudriavzevii*	Alcohol aroma and slight sour aroma	+

^1^The Closest relative was in corresponding with that of Fig. 1

^2^Weak ( +), intermediate (+ +), strong (+ + +)

Based on these results, nine strains (HN002, HN003, HN006, HN008, HN009, HN010, HN011, HN013, and HN014), which produced a pleasant aroma with moderate and strong intensity were selected for the follow-up experimental studies.

These nine strains were further examined for Chinese chestnuts wine fermentation, and each aroma profile of the obtained wine was measured by the SPME–GC–MS technique to further evaluate their performance in the accumulation of aroma compounds for the production of the Chinese chestnuts wine. Table 2 presents the list of all identified volatile compounds, which were mainly characterized by the following functional groups: 10 volatile acids (6.744–14.721 μg/mL), 14 esters (3.502–16.456 μg/mL), 11 alcohols (1.890–8.689 μg/mL), 6 aldehydes (0.309–3.974 μg/mL), 3 ketones (0.108–0.604 μg/mL), 3 *Alkanes* (0.373–1.188 μg/mL)*,* and, at a lower content, by 2 volatile phenols (0–0.409 μg/mL).

**Table 2:** The volatile aroma compounds detected and measured in Chinese chestnut wines samples obtained by using various yeast strains

Number	Aroma compounds	Retention time (min)	Identification	Contents of volatile aroma compounds of 9 yeast strains/(μg/mL)								
				HN002	HN003	HN006	HN008	HN009	HN010	HN011	HN013	HN014
	*Volatile acids*											
AC1	Acetic acid	9.879	MS, RI	3.851	1.618	1.021	4.373	1.040	4.53	3.382	3.376	2.861
AC2	Propionic acid	12.377	MS, RI	1.012	1.358	0.641	1.043	0.458	1.89	0.289	1.112	0.326
AC3	Butyric acid	14.913	MS, RI	2.063	1.837	2.474	2.384	2.565	0.738	2.382	1.175	2.276
AC4	Hexanoic acid	16.314	MS, RI	4.198	2.541	1.241	1.885	1.770	0.497	1.585	2.519	1.112
AC5	Palmitic acid	34.615	MS, RI	1.213	1.098	0.674	1.142	0.454	1.831	0.762	1.041	0.785
AC6	Octanol acid	19.512	MS, RI	1.062	0.602	0.754	1.141	0.457	2.112	0.752	0.421	0.526
AC7	Oleic acid	37.021	MS, RI	0.243	0.121	ND	ND	ND	2.076	0.163	ND	0.207
AC8	Linoleic acid	36.047	MS, RI	0.158	0.125	ND	ND	ND	1.047	0.103	ND	0.137
AC9	2-methyl-butanoic acid	15.588	MS, RI	ND	ND	ND	ND	ND	ND	0.454	ND	0.210
AC10	nonanoic acid	26.761	MS, RI	ND	ND	ND	ND	ND	ND	ND	0.324	ND
	Σ			13.800	9.300	6.805	11.968	6.744	14.721	9.874	7.644	8.440
	*Esters*											
ES1	Ethyl acetate	4.032	MS, RI	5.009	2.754	2.524	5.123	2.187	6.014	3.612	3.894	3.047
ES2	Ethyl isobutanoat	5.567	MS, RI	1.232	0.469	0.118	0.954	0.214	0.707	ND	NA	ND
ES3	Ethyl butanoate	5.443	MS, RI	1.121	0.457	0.116	1.785	0.125	0.757	0.347	1.357	0.298
ES4	Isoamyl acetate	7.654	MS, RI	0.878	0.301	0.264	0.987	0.234	0.735	0.312	0.732	0.278
ES5	Ethyl caproate	5.620	MS, RI	0.187	0.257	ND	0.312	ND	0.489	0.137	0.127	0.203
ES6	3-Methyl-1-butanol acetate	8.943	MS, RI	2.642	0.114	ND	0.914	ND	1.863	ND	ND	ND
ES7	Isopentyl hexanoate	9.753	MS, RI	0.952	0.147	ND	0.321	ND	ND	0.214	0.213	0.157
ES8	Phenethyl acetate	11.573	MS, RI	0.203	0.124	0.124	0.257	0.113	0.464	ND	NA	ND
ES9	Hexyl acetate	6.525	MS, RI	0.524	0.684	0.289	0.389	0.202	0.497	0.217	0.337	0.275
ES10	Ethyl oenanthate	6.441	MS, RI	0.221	0.11	ND	0.234	ND	0.433	ND	ND	ND
ES11	Ethyl laurate	14.135	MS, RI	0.436	0.345	ND	0.257	ND	1.335	ND	ND	ND
ES12	Ethyl palmitate	28.268	MS, RI	0.641	0.835	0.465	0.738	0.427	1.427	0.547	1.218	0.62
ES13	Ethyl oleate	23.726	MS, RI	0.211	0.109	ND	ND	ND	0.914	0.123	ND	0.187
ES14	Ethyl linoleate	31.429	MS, RI	0.179	0.076	ND	ND	ND	0.721	0.101	ND	0.157
	Σ			14.436	6.782	3.900	12.271	3.502	16.456	5.610	7.878	5.222
	*Alcohols*											
AL1	Isoamyl alcohol	5.842	MS, RI	0.757	0.714	3.987	0.701	3.623	0.896	0.432	0.521	0.375
AL2	2,3-butanediol	13.399	MS, RI	0.524	0.364	0.914	0.412	0.625	0.687	0.153	0.178	0.161
AL3	2-methyl-1-propanol	10.321	MS, RI	0.847	0.557	1.851	0.496	1.787	1.287	0.277	0.242	0.225
AL4	3-ethoxy-1-propanol	1.358	MS, RI	0.467	0.432	0.754	0.384	0.624	0.988	ND	ND	ND
AL5	1-hexanol	7.281	MS, RI	0.747	0.128	0.323	0.687	0.214	0.887	0.118	0.217	0.132
AL6	1-octen-3-ol	11.11	MS, RI	0.234	0.357	0.653	0.312	0.41	0.301	0.596	0.441	0.588
AL7	Enanthol	11.223	MS, RI	0.216	0.165	0.294	0.202	0.272	0.237	ND	0.058	ND
AL8	Isooctanol	12.107	MS, RI	0.132	0.132	0.165	0.213	0.125	0.245	ND	ND	ND
AL9	Octanol	13.611	MS, RI	0.267	0.184	0.572	0.237	0.454	0.341	ND	ND	ND
AL10	1-nonanol	15.966	MS, RI	0.126	0.116	0.254	0.132	0.241	0.142	ND	ND	ND
AL11	Phenylethyl alcohol	20.685	MS, RI	1.878	0.783	0.354	1.567	0.314	2.453	0.314	1.235	0.231
	Σ			6.195	3.932	10.121	5.343	8.689	8.464	1.890	2.892	1.712
	*Aldehydes*											
AD1	Pentanal	18.153	MS, RI	0.321	0.204	0.723	0.214	0.663	0.282	ND	ND	ND
AD2	2-Heptenal	7.882	MS, RI	0.223	0.134	0.887	0.185	0.743	0.225	ND	ND	ND
AD3	2-undecenal	17.696	MS, RI	0.057	0.025	0.132	0.034	0.091	0.088	ND	ND	ND
AD4	Nonaldehyde	9.445	MS, RI	0.183	0.101	0.392	0.154	0.267	0.203	ND	ND	ND
AD5	Benzaldehyde	12.598	MS, RI	0.371	0.321	0.856	0.235	0.687	0.436	0.143	0.132	0.111
AD6	2-phenyl-2-butenal	21.557	MS, RI	0.187	0.314	0.984	0.301	0.452	0.469	0.231	0.123	0.198
	Σ			1.342	1.099	3.974	1.123	2.903	1.703	0.374	0.255	0.309
	*Ketones*											
KE1	2-octanone	6.962	MS, RI	0.207	0.112	0.154	0.187	0.108	0.238	ND	ND	ND
KE2	3-hydroxy-2-butanone (Acetoin)	9.611	MS, RI	0.201	0.215	ND	0.152	ND	0.209	0.474	0.437	0.468
KE3	2-nonanone	9.352	MS, RI	0.136	0.107	ND	0.113	ND	0.157	ND	ND	ND
	Σ			0.544	0.403	0.154	0.452	0.108	0.604	0.474	0.437	0.468
	*Alkanes*											
AK1	tetramethylethylene	4.321	MS, RI	0.292	0.085	0.138	0.216	0.154	0.387	0.126	0.198	0.134
AK2	2-methyl-2-butene	5.764	MS, RI	0.554	0.214	0.287	0.423	0.219	0.687	0.301	0.367	0.353
AK3	Phenylethylene	6.203	MS, RI	0.152	0.132	ND	0.086	ND	0.114	0.074	0.045	0.068
	Σ			0.998	0.431	0.425	0.725	0.373	1.188	0.501	0.610	0.555
	*Volatile phenols*											
PH1	4-Vinylphenol	20.312	MS, RI	0.181	0.113	ND	0.161	ND	0.185	0.164	0.089	0.128
PH2	4-Vinyl guaiacol	23.345	MS, RI	0.221	ND	ND	0.198	ND	0.224	0.216	0.134	0.178
	Σ			0.402	0.113	0	0.359	0	0.409	0.38	0.223	0.306

*MS* identification of compounds was by MS spectra, *RI* identification was by reference to RI values in the literature, *ND* not detected

Apart from presenting the overall aromatic profile of each tested strain, Table 2 also highlighted the differential behavior exhibited by strains belonging to the same species. We observed a variation in the total concentration of each type of volatile compounds based on different strains. The samples produced by non-*Saccharomyces* yeasts (HN002, HN003, HN008, HN010, HN011, HN013, and HN014) possessed higher amounts of volatile acids compared with those of *Saccharomyces* yeasts (HN006, HN009), especially the wine fermentation using the strain HN010 showed the highest concentration (15.721 μg/mL) of various acids, with the exception of butanoic acid, hexanoic acid, 2-methyl-butanoic acid, and nonanoic acid. Butanoic acid and hexanoic acid are generally present in the Chinese strong flavor liquor, and their high content can impart an unpleasant flavor to the wine (Aslankoohi et al. 2016). The presence of 2-methyl-butanoic acid in the strains HN011 and HN014 imparts cheese-like and sweet characteristics (Marycarmen et al. 2020), which were consistent with the results of our preliminary olfactory “sniff” test. Nonanoic acid, detected only in strain HN009, had green and fat characteristics (Aslankoohi et al. 2016). No significant difference was observed in the type and concentration of volatile acids between HN006 and HN009.

We observed a significant difference in the concentrations of esters in the wines produced using different cultures (Table 2). We found a significantly higher concentration of esters in the *non-Saccharomyces* culture wines compared with the *Saccharomyces* culture wines, which was probably attributed to its low lipase activity (Table 2). The highest concentration of esters (16.456 μg/mL) was found in the wine prepared using HN010, which was 3.22 and 3.70 times higher than that in the *Saccharomyces* yeast strain of HN006, HN009, respectively. Amongst all types of esters, the concentration of ethyl acetate was found to be the highest for each strain, ranging from 2.187 μg/mL (HN006) to 16.456 μg/mL (HN010). Ethyl esters, typically described to possess a “fruity and flower” aroma (Laura et al. 2019), were the largest ester-based compounds in the Chinese chestnut wine. We found extremely high amounts of ethyl acetate (sweet, fruity), phenylethyl acetate (rosy), and ethyl caproate (apple peel, fruity) in the Chinese chestnut wine produced by HN010 (Marycarmen et al. 2020), along with high-molecular-weight ethyl esters, such as ethyl laurate, ethyl palmitate, ethyl oleate, and ethyl linoleate, which were reported to promote the accumulation of the after-taste in the Chinese strong flavor liquor (Yan et al. 2019a, b).

Higher alcohols, produced by yeast, originate either from the degradation of branched-chain amino acids or directly from sugar fermentation. We found that the higher alcohols constituted the major quantitative component in *Saccharomyces* (HN006, HN009)-fermented chestnut wine samples, and were significantly higher than those obtained by *non-Saccharomyces*-fermented wines (Table 2). The wine samples brewed using the strain HN006 exhibited the highest concentration of total higher alcohols, which included isoamyl alcohol (3.987 μg/mL), 1-nonanol (0.254 μg/mL), 1-hexanol (0.323 μg/mL), 2,3-butanediol (0.914 μg/mL), isooctanol (0.165 μg/mL), 2-methyl-1-propanol (1.851 μg/mL), enanthol (0.294 μg/mL), 3-ethoxy-1-propanol (0.754 μg/mL), 1-octen-3-ol (0.653 μg/mL), octanol (0.572 μg/mL), and phenylethyl alcohol (0.354 μg/mL). Of these, isoamyl alcohol, 2,3-butanediol, and phenylethyl alcohol have previously been reported to be present in fermented alcoholic beverages as characteristic flavor compounds, such as Chinese strong flavor liquor (Yan et al. 2019a, b).

Aldehydes are produced from amino acids either by Strecker degradation or by transamination, followed by decarboxylation, and they can be easily reduced to alcohols. We observed that the *Saccharomyces*-fermented wines possessed a significantly higher concentration of aldehydes compared with the non*-Saccharomyces*-fermented wines. This trend was consistent with the trends of higher alcohols (Table 2).

Additionally, we detected three aldehydes in the Chinese chestnut wine samples, and they were found to be most abundant in the strain HN006. Among these, 2-octanone, which imparts a cream-like flavor to the wine body (Yan and Dong 2019), was found in the Chinese chestnut wine samples. Also, 2-nonanone, which has a fruity and flowery note (Yan and Dong 2019), was found in each chestnut wine sample fermented using strains HN005, HN006, HN007, and HN008.

We found the highest levels of alkanes (1.188 μg/mL) in the samples fermented by strain HN006, and they constituted tetramethylethylene (0.387 μg/mL), 2-methyl-2-butene (0.687 μg/mL), and phenylethylene (0.114 μg/mL). Additionally, two volatile phenols, namely 4-vinylphenol, and 4-vinylguaiacol, were also identified to be present in the highest amount in the samples with strain HN006, and are known to possess a characteristic spice-like aroma (Yan et al. 2019a, b).

**Fig. 2:**
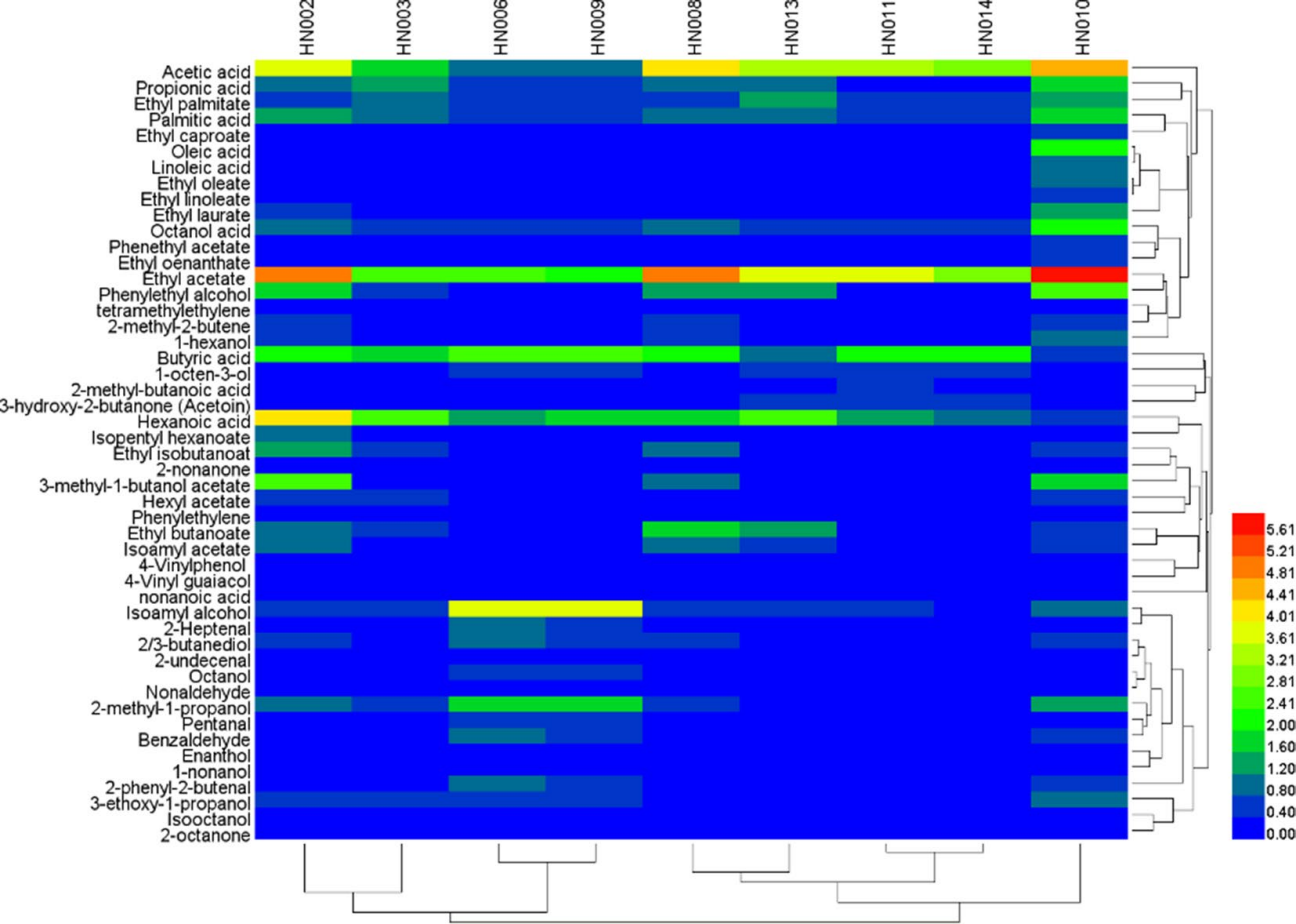
Heatmap illustrating the differences in the concentrations of volatile compounds in the samples of Chinese chestnut wine prepared with various yeast strains

We created a heatmap representation to allow a rapid visual assessment of the similarities and differences in the volatile profile of the strains between different samples. Additionally, the acids, alcohols, and esters represented the major functional groups for all the yeast strains (Fig. 2). Figure 2 shows the differences in the relative abundance of compounds among different strains, revealing the presence of different flavor profiles in different strains. For instance, the *Saccharomyces* strains (HN006, HN009), produced high levels of ethyl esters, isoamyl alcohol, and 2-methyl-1-propanol, along with the *non-Saccharomyces* strains (HN004 and HN010). Notably, the strain HN010 showed relatively larger amounts of volatile compounds compared with other strains, which indicated that it might produce wine with a more harmonious wine aroma. These results also suggested that different yeast strains, especially *non-Saccharomyces* yeasts, imparted a specific influence on wine flavor. However, their fermentation characteristics are not yet fully understood, and further research is required to identify the wine fermentation capacity of each strain.

### Testing for fermentation ability

**Fig. 3:**
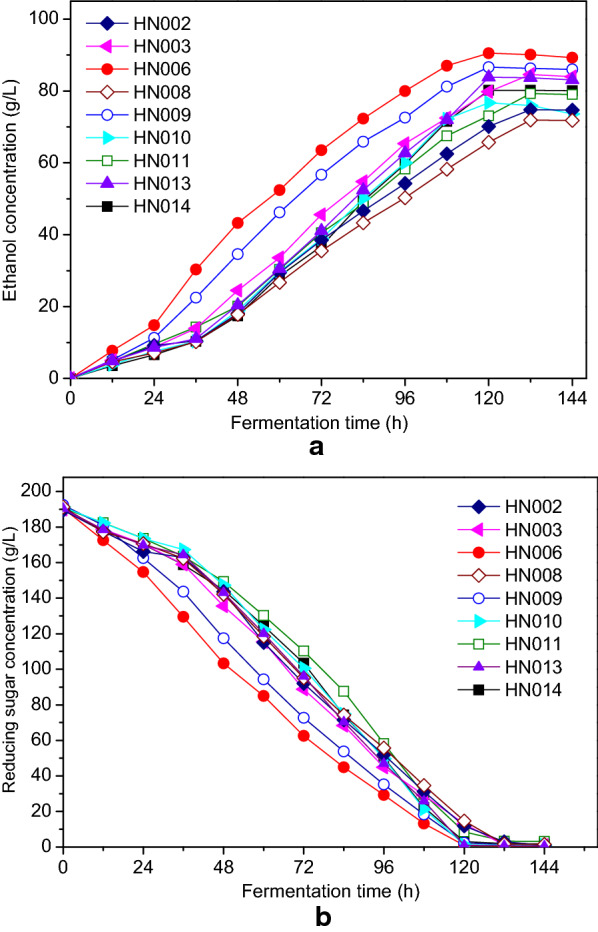
Fermentation profiles of Chinese chestnut wine with various yeast strains: **a** ethanol; **b** reducing sugar

We tested the fermentation performance of the obtained nine yeast strains to identify the optimal yeast strain for preparing the Chinese chestnut wine. Figure 3a, b show the effects of yeast strains on the concentrations of ethanol and reducing sugar during the fermentation of Chinese chestnut wine. We observed a slight increase in the concentration of ethanol for *Saccharomyces* strains (HN006, HN009) during the initial 24 h of fermentation, which increased significantly and reached the maximum value after 120 h, whereas the ethanol content produced by non*-Saccharomyces* strains (HN002, HN003, HN008, HN010, HN011, HN013, and HN014) got slowly accumulated throughout the fermentation, and their corresponding concentrations were lower than those produced by the *Saccharomyces* strains. Table 3 also summarizes the kinetic parameters of the ethanol fermentation with different strains. The strain HN006 generated the maximum concentration of ethanol (90.220 ± 0.874 g/L), had the highest ethanol productivity (0.752 ± 0.026 g/L/h), and sugar utilization (99.5%) after 120 h (Table 3). Also, this strain achieved the highest final ethanol yield of 0.467 ± 0.043 g/g. Thus, we observed that the strain HN006 showed the high fermentation performance of Chinese chestnut wine.

**Table 3:** Evaluation of kinetic parameters for Chinese chestnut wine by various yeast strains

Strain code	Initial reducing sugar concentration (g/L)	Fermentation time (h)	Maximum ethanol concentration (g/L)	Sugar utilization (%)	Ethanol yield (g/g)	Volumetric ethanol productivity (g/L/h)
HN002	189.5 ± 2.2	132	74.9 ± 2.0	96.0 ± 3.9	0.40 ± 0.02	0.57 ± 0.01
HN003	189.9 ± 2.0	132	84.4 ± 1.2	99.3 ± 3.19	0.45 ± 0.03	0.64 ± 0.02
HN006	191.6 ± 2.0	120	90.2 ± 0.9	99.500 ± 5.6	0.47 ± 0.04	0.75 ± 0.03
HN008	191.3 ± 2.0	132	71.9 ± 1.9	97.5 ± 2.6	0.38 ± 0.02	0.55 ± 0.03
HN009	192.4 ± 3.4	120	86.6 ± 1.0	98.8 ± 4.8	0.46 ± 0.04	0.72 ± 0.02
HN010	191.2 ± 3.7	120	76.7 ± 1.1	98.5 ± 4.6	0.40 ± 0.03	0.64 ± 0.02
HN011	190.7 ± 3.1	132	79.3 ± 1.0	95.5 ± 3.5	0.42 ± 0.03	0.66 ± 0.02
HN013	190.4 ± 2.5	120	83.8 ± 1.4	98.9 ± 4.7	0.44 ± 0.03	0.70 ± 0.03
HN014	190.6 ± 2.2	120	80.2 ± 1.8	98.4 ± 2.4	0.43 ± 0.03	0.67 ± 0.02

### Sensory evaluation

A sensory evaluation was performed to identify the yeast strain that had the potential to produce a pleasant tasting wine. Sensory characteristics are vital markers to evaluate the wine quality, amongst which sensory appraisal is a commonly accepted method. A significant difference was observed in different quality attributes, such as aroma, after taste, wine taste, mouthfeel, and overall quality (Additional file 1: Figure S3). Among these wine samples, the wine prepared using strain HN010 was superior to those with other strains based on its highest scores for taste (7.37), aroma (7.76), mouth feel (7.64), after taste (6.90), and overall acceptability (7.12), which agreed with the result of aromatic profile of the volatile compounds determined by SPME–GC–MS. The *Saccharomyces* strains (HN006, HN009) received the lowest scores in the sensory analysis. Thus, this test identified and verified that the taste-profile of the Chinese chestnut wine made using the yeast strain HN010 was appreciated by the testers and had an affirmative effect on the overall sensory features.

Thus, the non-*Saccharomyces* strain, HN010, showed the highest amounts of volatile compounds and best sensory characteristics but with poor ethanol fermentation capacity, and the *Saccharomyces* strain, HN006, showed the highest efficiency in ethanol production and consumption of reducing sugar. Therefore, both these yeast strains were selected to be used as mixed inoculum for producing an improved version of the Chinese chestnut wine. Both strains were deposited in China Center of Industrial Culture Collection (CICC) and assigned the number of CICC1035 (HN006) and CICC1783 (HN010), respectively.

### Optimization inoculation sizes for chestnut whiskey brewing

We tested different inoculation volumes of strains HN006 and the strain HN010 to inoculate the fermentation mash at the beginning of the fermentation process of the Chinese chestnut wine, and their inoculums were pre-cultured separately as specified in Sect. 2.2. Table 4 lists the effects of the mixed inoculation volumes with different ratios of strains HN006 and HN010 on the concentration of ethanol and overall sensory scores. We observed an increase in the concentration of ethanol with an increase in the overall combined inoculation volume and reached a maximum value at 6% (v/v). Further increase in the inoculation volume resulted in a decrease in ethanol concentration. There was no significant difference in ethanol concentration for different inoculation ratios of HN006 and HN010 at an inoculation size of 5%. Further modification in the size and ratio of this inoculation resulted in a decreased ethanol production. As for the overall sensory scores, it showed an increasing trend with an increase in the inoculation ratio of the strains HN010 to strain HN006 at various inoculation volumes below 6%; however, there was no significant difference in the overall sensory scores for various inoculation ratios as the inculcation size reached up to 12% (v/v). The highest overall sensory score was also achieved at the mixed inoculation size of 6% (v/v) and a HN006 to HN010 inoculation ratio of 1: 2 (v/v). Thus, we used this inoculum size in further studies.

**Table 4:** Effect of different inoculum sizes and ratios of strain HN006 and strain HN010 on production of Chinese chestnut wine after 6 days of co-culture fermentation

Inoculum size (%)	HN006: HN010 (v/v)	Initial reducing sugar concentration (g/L)	Ethanol concentration (g/L)	Ethanol yield (g/g)	Overall sensory scores
3	2: 1	157.4 ± 3.7	65.8 ± 3.7	0.44 ± 0.01	7.2 ± 0.2
	1: 1	156.9 ± 2.9	62.8 ± 2.9	0.42 ± 0.02	7.3 ± 0.2
	1: 2	157.2 ± 2.6	68.6 ± 2.6	0.40 ± 0.02	7.4 ± 0.2
6	2: 1	152.7 ± 2.3	71.0 ± 3.2	0.47 ± 0.03	7.3 ± 0.2
	1: 1	152.7 ± 3.5	70.9 ± 3.5	0.46 ± 0.03	7.4 ± 0.2
	1: 2	152.5 ± 2.2	71.2 ± 2.2	0.47 ± 0.02	7.5 ± 0.2
9	2: 1	147.3 ± 2.6	60.6 ± 2.6	0.41 ± 0.02	7.4 ± 0.3
	1: 1	145.7 ± 3.3	54.7 ± 3.3	0.39 ± 0.02	7.5 ± 0.3
	1: 2	146.9 ± 2.9	55.9 ± 2.9	0.38 ± 0.03	7.5 ± 0.2
12	2: 1	142.8 ± 3.9	52.2 ± 3.9	0.37 ± 0.02	7.4 ± 0.2
	1: 1	143.3 ± 2.6	51.3 ± 2.6	0.36 ± 0.02	7.4 ± 0.2
	1: 2	142.3 ± 2.9	49.6 ± 2.9	0.35 ± 0.02	7.4 ± 0.2

± indicates standard derivation among replicates

### Optimization the ratios of different raw material for chestnut whiskey brewing

Table 5 illustrates the influence of raw materials with different ratios on the brewing of the Chinese chestnut whiskey. The ratio of chestnut whiskey to malt, and to glutinous rice in the medium had a crucial effect on the sensory score and the production of ethanol.

**Table 5:** Effect of different ratios of raw material on production of Chinese chestnut wine after 6 days of co-culture fermentation

Raw material ratio (Chinese chestnut: malt: glutinous rice, w: w: w)	Initial reducing sugar concentration (g/L)	Ethanol concentration (g/L)	Overall sensory scores
4: 4: 2	208.6 ± 3.5	85.5 ± 2.9	7.8 ± 0.5
4: 3: 3	203.5 ± 2.9	85.0 ± 3.5	7.6 ± 0.4
4: 2: 4	198.5 ± 3.8	86.0 ± 3.8	7.5 ± 0.2
5: 3: 2	188.7 ± 2.2	86.0 ± 2.5	8.2 ± 0.3
5: 2.5: 2.5	181.7 ± 2.2	84.3 ± 3.0	7.9 ± 0.5
5: 2: 3	178.2 ± 2.9	82.5 ± 2.9	7.8 ± 0.4
6: 3: 1	171.6 ± 3.5	80.6 ± 3.5	7.9 ± 0.5
6: 2: 2	164.5 ± 2.9	77.5 ± 2.9	7.8 ± 0.4
6: 1: 3	161.6 ± 3.8	75.6 ± 3.8	7.7 ± 0.3

The data presented in Table 5 revealed an increase in the initial reducing sugar concentration of the saccharified mash with the total amounts of malt and glutinous rice. The ethanol concentration and overall sensory scores increased as the proportion of malt and glutinous rice to Chinese chestnut increased, and both reached a maximum value at the ratio of 5:3:2 (Chinese chestnut: malt: glutinous rice). All multiple-grain fermentations were superior to single-grain fermentation of Chinese chestnut, as shown by a higher overall sensory score and the ethanol content. Table 5 demonstrated that the optimum ratio of chestnut whiskey to malt to glutinous rice, and to corn was 5:2:2:1 (w: w: w: w), which is justified by the highest corresponding value of the sensory score (8.210 ± 0.300) and highest ethanol concentration (86.021 ± 2.470). This value will be used for further testing.

### Optimization nitrogen nutrient additions for chestnut whiskey brewing

Table 6 demonstrates the effect of the addition of various amounts of nitrogen nutrients on the fermentation of the Chinese chestnut wine. We observed an increase in the production of ethanol with an increase in the supplementation amounts of yeast extract. We obtained the maximum concentration of ethanol (92.587 ± 3.872 g/L) was obtained at the yeast extract concentration of 6 g/L, compared with the fermentation without the addition. As for the overall sensory scores of the obtained wine, we observed a gradual increase with an increase in the amounts of yeast extract added and reached the maximum of 8.910 ± 0.300 when 6 g/L yeast extract was supplemented in the fermentation system; however, there was no significant change with a further increase in the supplementation of the yeast extract. This phenomenon was attributed to the fact that the appropriate amount of yeast extract promoted aroma production, while an excess induced the growth of the strain but was disadvantageous to the formation of the end product. Thus, both the productivity and the sensory characteristics were improved at 6 g/L of yeast extract (Table 6). Further investigation is required to study the impact of the supplementation of nitrogen on the metabolic pathways involved in producing and regulating aroma, as well as to understand how nitrogen affects the process of yeast metabolism in alcoholic fermentation, which will promote an improved utilization of this nutrient to produce volatile compounds.

**Table 6:** Effect of different supplementation amounts of nitrogen nutrient on production of Chinese chestnut wine after 6 days of co-culture fermentation

Supplementation amounts of yeast extract (g/L)	Initial reducing sugar concentration (g/L)	Ethanol concentration (g/L)	Overall sensory scores
0	189.5 ± 4.0	85.7 ± 2.2	8.1 ± 0.4
2	188.6 ± 4.2	88.6 ± 3.5	8.3 ± 0.5
4	190.9 ± 3.6	90.7 ± 3.9	8.6 ± 0.4
6	189.6 ± 4.1	92.6 ± 3.9	8.9 ± 0.3
8	191.6 ± 3.9	86.8 ± 2.2	8.8 ± 0.5
10	190.9 ± 4.2	78.2 ± 2.8	8.7 ± 0.6

### Pilot-scale fermentation

After the completion of the fermentation process at the laboratory scale, the optimized parameters were tested in a scaled-up system of 1000 L reactor to investigate the possibility of industrializing the production of the Chinese chestnut wine. The system was used for producing three batches of Chinese chestnut fermented wine, with an agitation speed of 100 rpm and at a temperature of 30 °C.

**Fig. 4:**
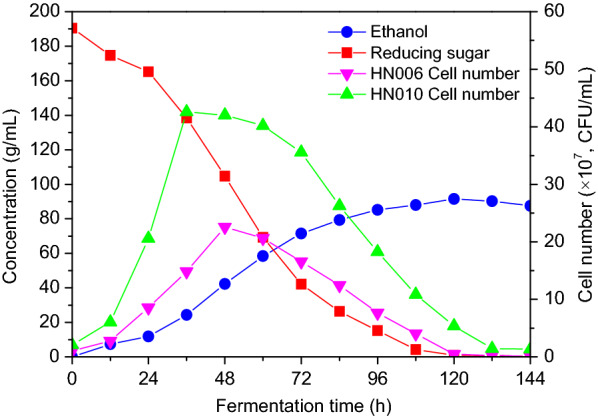
1000L fermentation profiles of Chinese chestnut wine with co-culture of strain HN006 and strain HN010

The Fig. 4 demonstrates the typical profiles of total cell growth, reducing sugar consumption and ethanol formation over time in the 1000 L bioreactor. After the initial 12-h lag phase, we observed an increase in the process of both reducing sugar consumption and ethanol production. The maximal growth for strain HN006 (2.065 × 10^8^ CFU/mL) and the strain HN010 (4.201 × 10^8^ CFU/mL) was observed after 48 h and 36 h of fermentation, respectively. The highest concentration of ethanol was 190.56 g/L, with a corresponding yield of 0.487 g/g. Over a 3-d period, we successfully completed three runs using the co-culture fermentation. In these three experiments, we observed the key fermentation parameters: final ethanol concentration, volumetric ethanol productivity, and ethanol yield to be consistent. The fermentation performance was successfully demonstrated in the 1000 L pilot-scale bioreactor and would be suitable for the industrial co-culture fermentation Chinese chestnut wine.

## Discussion

The flavour of distilled wine depends not only on the raw materials and manufacturing technique, but also on the characteristics of yeast and other microorganisms (Alicia et al. 2018). Yeasts are important and can be detected during the whole manufacturing process. Not only do they directly determine the fermentation rate, but they can also transform nutrients into a wide variety of volatile flavour compounds (Ayşe et al. 2018; Wang et al. 2019). Therefore, yeasts are one of the key factors affecting the flavour type and product quality of whiskey (Shi et al. 2019; Wu et al. 2012).

At present, most whiskeys are fermented by *Saccharomyces cerevisiae*. Although the single fermentation mode by *Saccharomyces cerevisiae* obtains high alcohol content, but it has the phenomenon of homogenization of whiskey flavor. Recently years, a large number of studies have shown that non-*Saccharomyces cerevisiae* can produce alcohols, esters, acids, alkanes, aromatic hydrocarbons, ketones and other metabolites in the fermentation process, which can enhance the flavour and enrich the taste of the wine, however, it normally showed poor ethanol fermentation capacity. Nowadays, co-fermentation of selected non-Saccharomyces yeasts with *S. cerevisiae* received more and more acceptance to avoid the defects of pure fermentation and spontaneous fermentation (Luan et al. 2018). Remarkably, the application of mixed cultures of *Saccharomyces cerevisiae* and non-*Saccharomyces cerevisiae* has been reported to make important contributions to the formation of aroma substances, and also with high efficiency of ethanol production (Yan et al. 2019a, b). Additionally, the use of different species of yeast has proved to improve the percieved flavour in wine (Alicia et al. 2018). Therefore, the selection of yeast strains is critical for the formation of wine aroma, which is one of the key attributes affecting consumer acceptance (Wang et al. 2019). Yuan et al. (2019) selected one excellent non-*Saccharomyces cerevisiae* strain from 6 aroma-producing yeast strains and co-cultured with *Saccharomyces cerevisiae* for Yali fruit wine fermentation and the results demonstrated that the mixed fermentation helped to enhance the aroma and improve the complexity and characteristics of the wine.

In present study, a novel whiskey using Chinese chestnut as the main raw material was developed. First, we isolated the yeast strains and investigated their potential to produce effective Chinese chestnut wine, and the results demonstrated that the strain HN010, which showed the maximum production of the volatile compounds and the best sensory characteristics, along with the strain HN006 that possessed the highest efficiency in ethanol production were adopted as the most promising yeasts as they combined satisfactory Chinese chestnut wine fermentation with an interesting flavor profile. Further optimization of the Chinese chestnut wine fermentation using a co-culture of strain HN006 and strain HN010 demonstrated that the optimum conditions for chestnut wine production were achieved at a mixed inoculum volume of 6% (v/v) with an HN006/HN010 inoculation ratio of 1:2 (v/v), a raw materials ratio of 5:3:2 (chestnut whiskey: malt: glutinous rice), and a yeast extract concentration of 6 g/L. Additionally, a scaled-up fermentation of the Chinese chestnut wine was successfully carried out in a 1000 L pilot-scale system. This study is the first report to explore the fermentation of Chinese chestnut wine using a mixed culture system. The co-culture of strain HN006 and strain HN010 showed potential application in the Chinese chestnut wine fermentation process. Additionally, the methods used in this study could provide a generalized experimental scheme to initiate large-scale studies for the identification of useful microbes for the fermentation of wine as well as other food products.

## Supplementary Information

**Additional file 1: Figure S1. **The manufacturing process of Chinese chestnut whiskey raw wine. **Figure S2.** The layout of the 1000 L Chinese chestnuts wine fermentation pilot plant. 1, Soaking rice cans; 2, Steamed rice cooker; 3, Liquefied tank; 4, Pump; 5, Malt miller; 6, Saccharifying tank; 7, Pump; 8,Filter tank; 9, Pump; 10, Plate heat exchanger; 11, 10 L seed preparation reactor; 12, Pump; 13, 100L seed preparation reactor; 14, Pump; 15, 1000 L reactor; 16, Pump. **Figure S3.** Sensory evaluation of Chinese chestnut wine samples by different yeast strains.

## Data Availability

Please contact author for data requests.
