# Supplementary material for: Development of Chinese chestnut whiskey: yeast strains isolation, fermentation system optimization, and scale-up fermentation
Source: AMB Express. 2021 Jan 11;11:17. doi: 10.1186/s13568-020-01175-4 (PMC7801535; doi:10.1186/s13568-020-01175-4)
Supplement: Supplementary file 1 — Additional file 1: Figure S1. The manufacturing process of Chinese chestnut whiskey raw wine. Figure S2. The layout of the 1000 L Chinese chestnuts wine fermentation pilot plant. 1, Soaking rice cans; 2, Steamed rice cooker; 3, Liquefied tank; 4, Pump; 5, Malt miller; 6, Saccharifying tank; 7, Pump; 8,Filter tank; 9, Pump; 10, Plate heat exchanger; 11, 10 L seed preparation reactor; 12, Pump; 13, 100L seed preparation reactor; 14, Pump; 15, 1000 L reactor; 16, Pump. Figure S3. Sensory evaluation of Chinese chestnut wine samples by different yeast strains. [file 13568_2020_1175_MOESM1_ESM.doc]

**Supplementary materials**


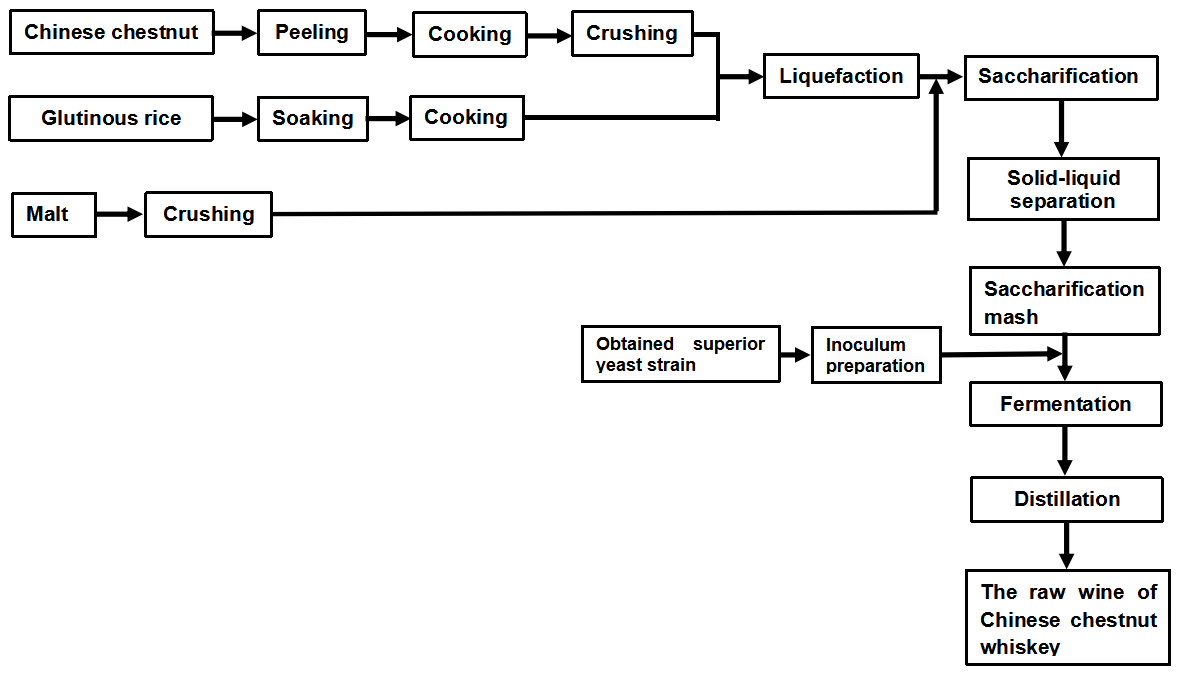


Fig. S1 The manufacturing process of Chinese chestnut whiskey raw wine

Fig. S2 The layout of the 1000 L Chinese chestnuts wine fermentation pilot plant. 1, Soaking rice cans; 2, Steamed rice cooker; 3, Liquefied tank; 4, Pump; 5, Malt miller; 6, Saccharifying tank; 7, Pump; 8,Filter tank; 9, Pump; 10, Plate heat exchanger; 11, 10 L seed preparation reactor; 12, Pump; 13, 100L seed preparation reactor; 14, Pump; 15, 1000 L reactor; 16, Pump.


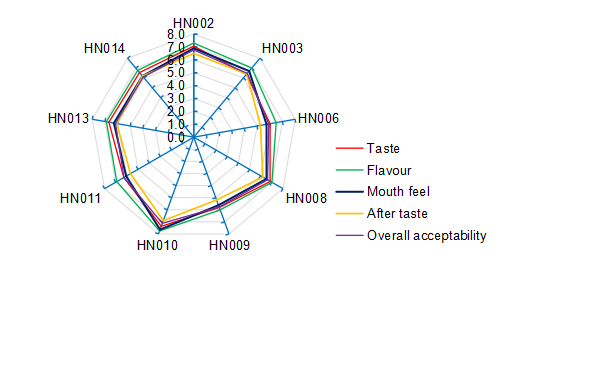


Fig. S3 Sensory evaluation of Chinese chestnut wine samples by different yeast strains
